# Studies on Transcriptional Incorporation of 5’-N-Triphosphates of 5’-Amino-5’-Deoxyribonucleosides

**DOI:** 10.1371/journal.pone.0148282

**Published:** 2016-02-01

**Authors:** Weronika Kotkowiak, Anna Pasternak, Ryszard Kierzek

**Affiliations:** Institute of Bioorganic Chemistry, Polish Academy of Sciences, Poznan, Poland; Florida Atlantic University, UNITED STATES

## Abstract

In this study, several RNA polymerases were used for the first time to examine the possibility of transcriptional incorporation of 5’-N-triphosphates of 5’-amino-5’-deoxyribonucleosides (5’NH NTPs). The T3, T7, Sp6 and T7 Y639F RNA polymerases were employed to show that the full-length transcript cannot be synthesized. The results suggest that the application of 5’NH NTPs could decrease transcription reaction rates. What is more, the modification of transcription conditions had no influence on the rate of 5’NH NTPs incorporation. Based on experimental data it is postulated that 5’NH NTPs can be used as potential transcription inhibitors. Our findings expand the knowledge on suitable uses of the 5’-N-triphosphates of 5’-amino-5’-deoxyribonucleoside and the exact mechanism of transcriptional inhibition.

## Introduction

During transcription, DNA-dependent RNA polymerases incorporate 5’-O-triphosphates of ribonucleosides into newly synthesized RNA. Previous studies have shown that also some modified derivatives such as 5’-O-triphosphates of 2’-fluoro- and 2’-amino-2’-deoxyribonucleosides [1] as well as 5’-(α-P-seleno)-triphosphates of ribonucleosides [2] are substrates for various RNA polymerases. Nevertheless, the incorporation efficiencies of modified triphosphates can differ depending on the type of bacteriophage RNA polymerases, such as T7 (RNAP T7), T3 (RNAP T3) and Sp6 (RNAP Sp6). T7 RNA polymerase exhibits the widest substrate tolerance, and replacement of the Y639F and H784A residues in the enzyme significantly enhances this tolerance. Both mutants of T7 RNA polymerase can incorporate 5’-O-triphosphates of 2’-O-methyl ribonucleosides and 2’-azido-2’-deoxyribonucleosides into the newly synthesized RNA [3–5].

The 5’-N-triphosphates of 5’-amino-2’,5’-dideoxyribonucleosides (5’NH dNTPs) were first described by Letsinger [6]. These nucleotide analogs have the 5’-hydroxyl group of the ribose replaced by an amino group. This substitution causes an increase in the chemical reactivity of 5’NH dNTPs and makes them more susceptible to acidic hydrolysis [6, 7]. Furthermore, 5’NH dNTPs have been reported to be substrates for DNA-dependent DNA polymerase and T4 DNA ligase [6–8]. These interesting properties of 5’NH dNTPs were applied in new methods of analysis of single-nucleotide polymorphisms (SNPs), short tandem repeat polymorphisms (STRs) [9] and genome sequence assembly [10, 11]. Moreover, biological studies have indicated that 5’NH dNTPs represent potent viral and bacterial inhibitors [12, 13]. The chemical synthesis of protected phosphoramidites of 5’-amino-2’,5’-dideoxyribonucleosides allowed to obtain oligonucleotides bearing phosphoramidate bonds, which exhibited improved resistance toward nucleolytic enzymes [14, 15].

The first application of the 5’-amino-5’-deoxyribonucleoside derivatives to RNA transcription was described by Szostak in 1998. The researchers incorporated 5’-amino-5’-deoxyguanosine as the first nucleoside in a transcript using DNA-dependent T7 RNA polymerase [16]. The amino group could be further modified in post-transcriptional reactions. The transcription rate was low due to the poor solubility of 5’-amino-5’-deoxyguanosine in aqueous buffers, but phosphorylation of the 5’-amino-5’-deoxyguanosine prior to transcription significantly improved its yield [17]. However, only the incorporation of the protected 5’-amino-5’-deoxyguanosine at the 5’-end of oligonucleotides by phosphoramidite approach provided a large pool of oligonucleotides for biological studies. These oligonucleotides are resistant to nucleolytic degradation, though they maintain RNAi-inducing activities [18].

The possibility of transcriptional incorporation of the 5’-N-triphosphates of 5’-amino-5’-deoxyribonucleosides (5’NH NTPs) has not yet been examined. Herein, we show for the first time the wide range of studies, which demonstrated that 5’NH NTPs inhibit DNA-dependent RNA polymerases. The 5’NH NTPs inhibited T7 RNA polymerase and the Y693F mutant, the bacteriophage T3 RNA polymerase and the bacteriophage Sp6 RNA polymerase.

## Experimental

### Synthesis of 5’-N-triphosphates of 5’-amino-5’-deoxyribonucleosides (10)

The synthesis of 5’-N-triphosphates of 5’-amino-5’-deoxyribonucleoside was performed according to a previously described method with a minor modification [10]. The reaction mixture consisted of 0.01 mmol 5’-amino-5’-deoxyribonucleoside, 0.05 mmol trisodium trimetaphosphate, 0.5 mM aqueous Tris-base (pH 11) and RNase-free water. The final volume of the reaction was 400 μl. The resulting solution was incubated at room temperature for 7 days. The products of the reaction were purified by thin-layer chromatography (TLC) and high-performance liquid chromatography (HPLC). The 5’-N-triphosphates were precipitated as sodium salt with 1% sodium perchlorate in acetone. The resulting precipitates were centrifuged, washed a few times with acetone and dried. Finally, 5’-N-triphosphates of 5’-amino-5’-deoxyribonucleosides were dissolved in RNase-free water and 10 mM stock solutions were prepared. The purity of all 5’NH NTPs stock solutions was verified via HPLC analysis (S1A–S1D Fig) and T4 polynucleotide kinase phosphorylation reaction (T4 PNK, S2 Fig) indicating lack of any contaminants which could affect polymerases activities.

### Chemical synthesis of oligonucleotides

All oligonucleotides were synthesized on an automated RNA/DNA synthesizer using standard phosphoramidite chemistry [19, 20]. The details of deprotection and purification of oligonucleotides were described previously [21–23]. The purity of all oligonucleotides was determined to be greater than 95% and the composition was confirmed by MALDI-TOF mass spectrometry.

### Transcription reaction

The transcription reactions were performed using commercially available DNA-dependent RNA polymerases: T3 (Promega), Sp6 (Promega), T7 (Ambion) and T7 Y639F mutant (Epicenter). The template was double-stranded, linear oligomer (160 bp, S1 Table) coding 5S ribosomal RNA (120 nt) from *Escherichia coli* and 8 μg were added per reaction. The reaction buffers consisted of 40 mM Tris-acetate (pH 8), 5 mM DTT, 1 mM Na_2_EDTA, 10 mM Mg-acetate, 0.5 mM MnCl_2_, 8 mM spermidine (marked as buffer BT1) or 40 mM Tris-acetate (pH 8), 5 mM DTT, 1 mM Na_2_EDTA, 10 mM Mg-acetate, 0.5 mM MnCl_2_, 13 mM spermine (marked as buffer BT2) or 40 mM Tris-acetate (pH 8), 5 mM DTT, 1 mM Na_2_EDTA, 10 mM Mg-acetate, 0.5 mM MnCl_2_, 1.6 mM spermine (marked as buffer BT3) or 40 mM CHES (pH 9), 10 mM DTT, 35 mM MgCl_2_, 0.01% Triton X-100, 1 mM spermidine (marked as buffer BT4). The standard buffers provided by enzyme suppliers were also tested. The 2 mM or 1 mM solutions of ATP, CTP, GTP, UTP and the 2 mM or 2.5 mM solutions of 5’NH ATP, 5’NH CTP, 5’NH GTP, 5’NH UTP were used. The 1 μl of 100 mM DTT, 1 μl of RNasin (Promega), 1.5 μl of DNA-dependent RNA polymerase (30 U) and RNase-free water (adjusted to 10 μl) were added to the reaction mixture. The reaction mixture was incubated overnight at 37°C. The reaction was quenched by adding 1 μL of TURBO DNase (Ambion) and incubated at 37°C for 15 min. Subsequently, overnight ethanol precipitation at 4°C, followed by centrifugation, was performed. The transcription product was 3’-^32^P labeled using [ɣ-^32^P]-pCp and T4 RNA ligase (Thermo Scientific) in accordance with its original protocol. Next, the resultant reaction mixture was mixed with 5 μl of a 95% deionized formamide solution containing xylene cyanol, bromophenol blue and orange G. The samples were denatured at 90°C for 3 min, then cooled on ice and loaded on a 12% denaturing polyacrylamide gel prepared in 1x TBE buffer. The electrophoresis was performed at 20 W for 4 h at room temperature. The resulting gel was imaged by storage phosphor technology using a Fuji Phosphorimager and MultiGauge Analysis Software.

### Primer extension assay

The primers were 5’-^32^P labeled by [γ-^32^P]-ATP using T4 polynucleotide kinase (EURx) in accordance with the manufacturer’s protocol. The 4 pmole of each 5’-end labeled primers was mixed with 4 pmole of the appropriate template. The samples were denatured for 7 min at 70°C and then slowly cooled to room temperature. After renaturing, 1 μl of T7 10X reaction buffer (Epicenter), 1 μl of 100 mM DTT (Epicenter), 0.25 μl of RNasin^®^ Ribonuclease Inhibitor (Promega), 1 μl of T7 Enzyme Mix (Epicenter), and 0.5 μl of ultrapure water were added (the total reaction volume was 10 μl), followed by 10 min incubation at room temperature. Primer extension polymerization was carried out by the addition of 0.5 μl of 1 mM solution of modified 5’NH NTP or natural NTP for 45 min at 37°C. The reaction was quenched by overnight ethanol precipitation at 4°C, followed by centrifugation and the subsequent addition of 5 μl of ultrapure water and 5 μl of urea solution containing xylene cyanol, bromophenol blue and orange G. The samples were loaded onto a 20% denaturing polyacrylamide gel prepared in 1x TBE buffer. The electrophoresis was performed at 20 W for 3 h at room temperature. The resulting gel was imaged by storage phosphor technology using a Fuji Phosphorimager and MultiGauge Analysis Software.

### T4 PNK phosphorylation reaction

The phosphorylation reaction mixture consisted of 300 pmol of RNA primer, 5 μl of 10 mM 5’NH NTP or natural NTP, 5 μl T4 PNK 10x reaction buffer (EUR_x_), 10U of T4 PNK enzyme mix (EUR_x_), RNase-free water (adjusted to 50 μl). The reaction was carried out for 30 min at 37°C and quenched by overnight ethanol precipitation at 4°C, followed by centrifugation and the subsequent addition of 5 μl of ultrapure water and 5 μl of urea solution containing xylene cyanol. The samples were loaded onto a 20% denaturing polyacrylamide gel prepared in 1x TBE buffer. The electrophoresis was performed at 20 W for 1 h at room temperature. The resulting gel was subsequently stained with SYBR GOLD and imaged using a Fuji Phosphorimager and MultiGauge Analysis Software. The 5’-phosphorylated oligonucleotide migrated faster than the 5’-OH variant owing to its extra negative charge, as previously reported [24, 25].

### Acidic cleavage

The cleavage reaction was performed by mixing 2 μl of 5% (v/v) aqueous acetic acid with 8 μl of primer extension reaction product and incubated at 37°C for 20 min. The reaction was ended by cooling on dry ice. The 100 μl of H_2_O was added, followed by evaporation to dryness using a Speedvac. Next, the resultant pellet was dissolved in 5 μl H_2_O and mixed with 5 μl of a 95% deionized formamide solution containing xylene cyanol, bromophenol blue and orange G. The samples were denatured at 90°C for 3 min, then cooled on ice and loaded on a 12% denaturing polyacrylamide gel prepared in 1x TBE buffer. The electrophoresis was performed at 20 W for 4 h at room temperature. The resulting gel was imaged by storage phosphor technology using a Fuji Phosphorimager and MultiGauge Analysis Software.

## Results and Discussion

**Fig 1:**
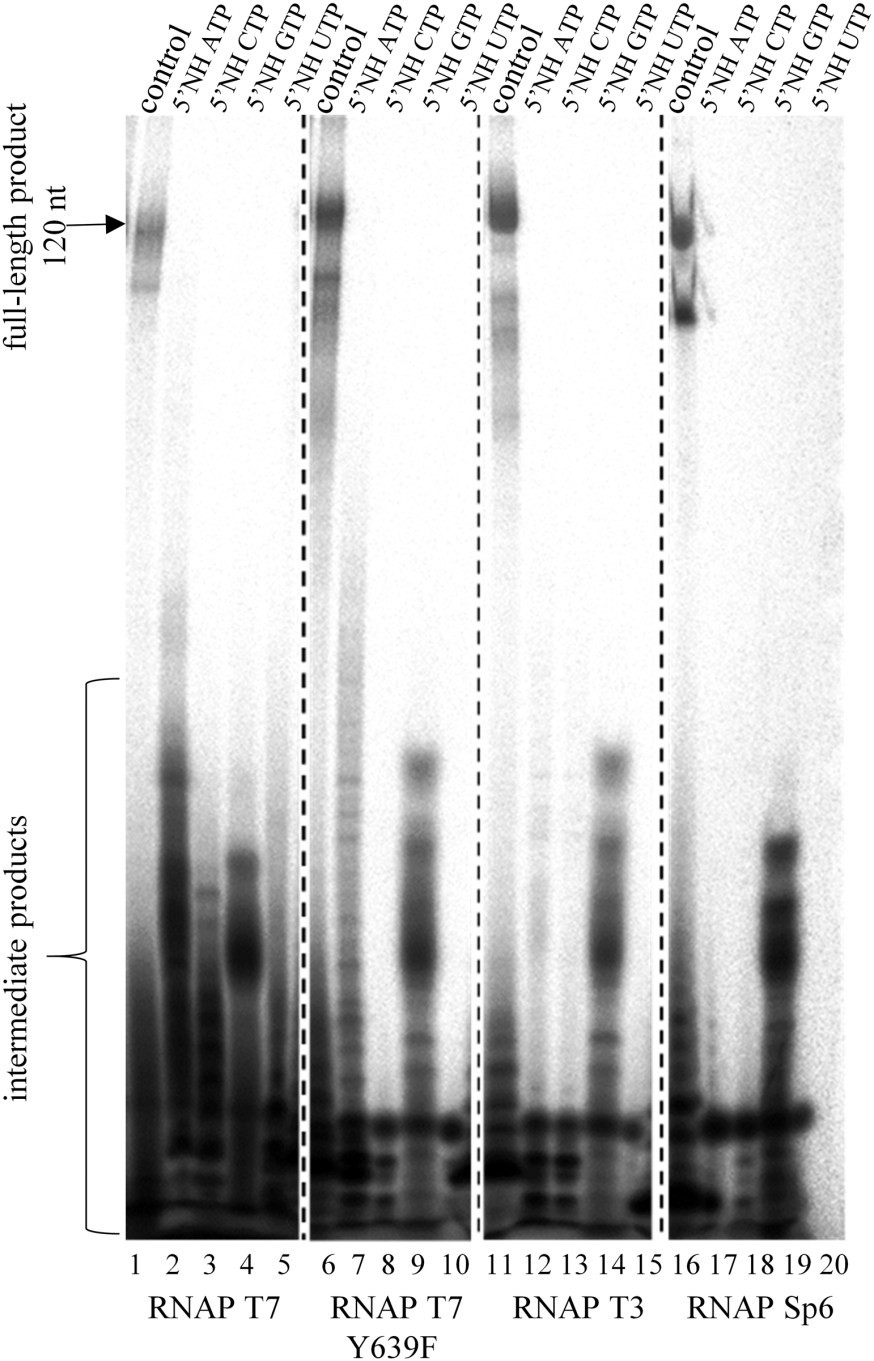
Transcription reaction. Transcription with 5S rRNA DNA templates (oligomer O1 and O2, S1 Table) using four natural NTPs (lanes 1, 6, 11, 16) or 5’NH ATP and 3 natural NTPs (lanes 2, 7, 12, 17) or 5’NH CTP and 3 natural NTPs (lanes 3, 8, 13, 18) or 5’NH GTP and 3 natural NTPs (lanes 4, 9, 14, 19) or 5’NH UTP and 3 natural NTPs (lanes 5, 10, 15, 20). Lanes 1–5 –RNAP T7. Lanes 6–10 –RNAP T7 Y639F. Lanes 11–15 –RNAP T3. Lanes 16–20 –RNAP Sp6. The gel is a representative of three replicate experiments.

Initially, we tested the ability of four bacteriophage RNA polymerases: T7, T7 Y693F mutant, T3 and Sp6 to incorporate 5’NH NTPs into a transcript. The reactions were run using 5S rRNA linear DNA-templates. For each reaction, the template contained the promoter sequence specific for the respective RNA polymerase, and the buffer and conditions were those recommended by the respective polymerase suppliers. The reaction mixture also consisted of three natural NTPs and one of 5’NH UTP, 5’NH CTP, 5’NH GTP or 5’NH ATP. The transcription results showed that the full-length transcript cannot be obtained (Fig 1). Nevertheless, when T7 RNA polymerase was used, we observed shorter, intermediate products in all four reaction variants (Fig 1, lanes 2–5). The Y639F T7 RNA polymerase mutant and the T3 RNA polymerase reactions showed intermediate products in the 5’NH ATP and 5’NH GTP reaction mixtures (Fig 1, lanes 7, 9, 12 and 14). When the transcription reaction was conducted with the Sp6 RNA polymerase, the intermediate product was detected only in the 5’NH GTP mixture (Fig 1, line 19). It was observed that modified nucleotides should be avoided in the first 10 nucleotides of transcript sequence [26–29]. Since the 5’NH GMPs possess initiation properties [17] presumably only the presence of this modified nucleotides among first nucleotides of transcript could be tolerated and in consequence resulted in occurrence of intermediate product. Transcription initiation involves the recognition of a specific promotor at the binding domain and formation a closed complex that converts into an open complex. It was shown that GTP possesses the ability to promote open complex formation and to modulate the equilibrium between open and close forms. Furthermore, a higher affinity GTP binding site in T7 RNAP apart from classical substrate-binding site was found [30], that indicates the importance of GTP for the correct transcription reaction. Additionally, the crucial role of GTP in initiating process also manifests due the fact that T7 RNAP promoter sequence begins with guanosine. It was previously reported, that about fourteen nucleotide long transcript could be formed, if the reaction mixture contained only GTP [31]. This observation suggests that initiation of transcription presented herein was not inhibited due to presence of GTP or 5’NH GTP in reaction mixture. Moreover, the promoter sequences of SP6 and T3 RNAPs also contained the guanosine at the beginning of the transcript and initiation properties of GTP/5’NH GTP can also be a true for these enzymes.

To improve the transcription efficiency, the influence of transcription buffer components on 5’NH NTPs incorporation was also investigated. It was reported, that spermine (1.5–3 mM) and spermidine (>8 mM) can aggregate nucleic acids and hence increase the yield of transcription reactions [4]. Based on this assumption, four buffers (BT1, BT2, BT3 and BT4) containing 8 mM spermidine, 13 mM spermine, 1.6 mM spermine, or 1 mM spermidine, respectively, were tested. In addition to 10 mM magnesium acetate, 0.5 mM MnCl_2_ was also added to these buffers. It has been previously reported that the use of Mn^2+^ as a cofactor in transcription reactions is beneficial for modulating the substrate specificity of RNA polymerases [32]. Furthermore, substrate specificity is even more improved by including a combination of Mg^2+^ and Mn^2+^ in the reaction; thus, both ions were added in performed experiments [33]. In the presented studies, all four RNA polymerases failed to produce a full-length transcript; however, shorter intermediate products were observed (S3A–S3E Fig).

**Fig 2:**
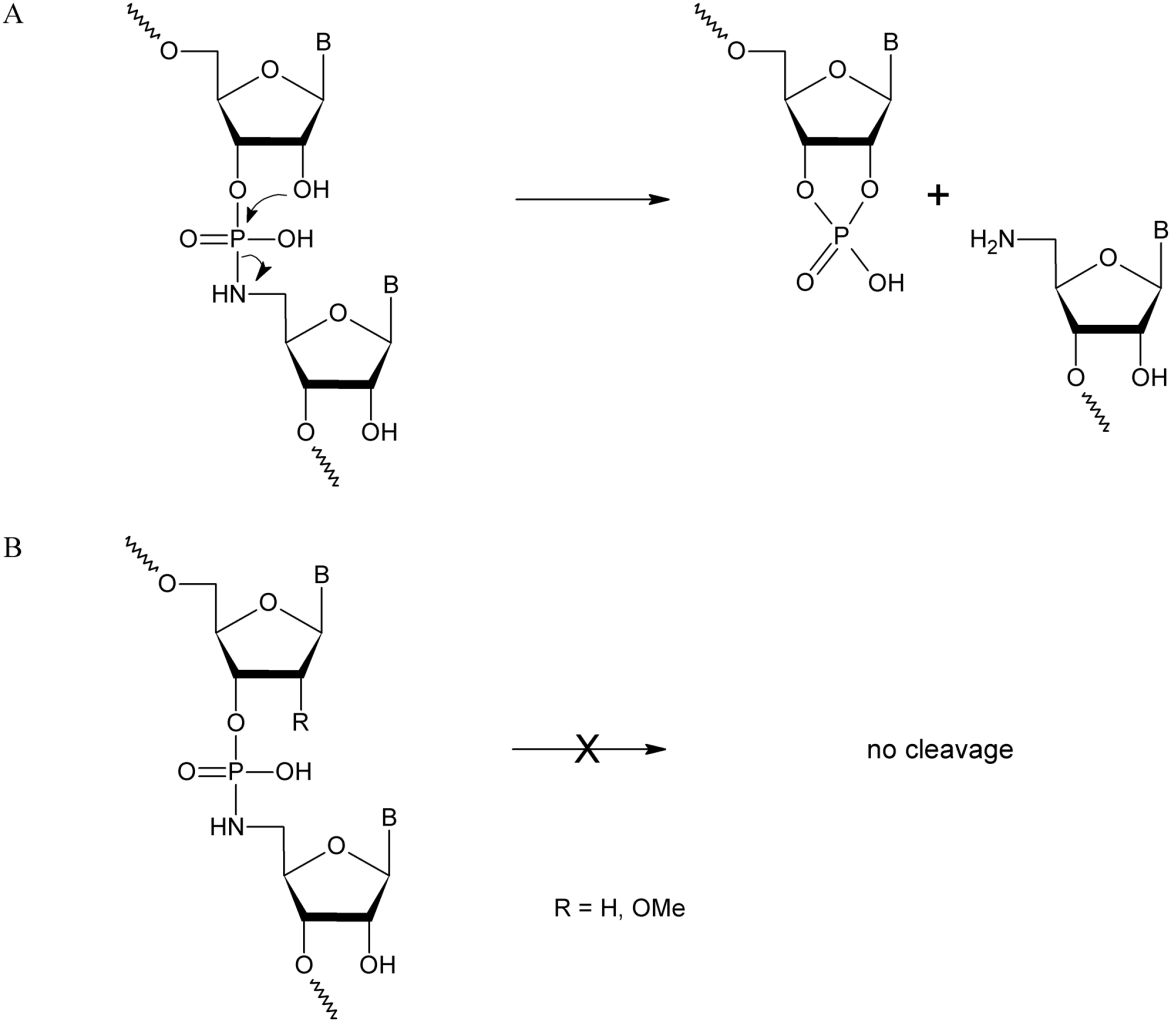
Mechanism of *in-line* internucleotide phosphoramidate bond cleavage. (A) Cleavage of internucleotide bond proceeds by an *in-line* mechanism as indicated by arrows, wherein the nucleophilic 2’-hydroxyl group attacks the phosphorus atom, generating 5’NH_2_ as a leaving group and 2’,3’-cyclic phosphate termini. (B) The presence of weak nucleophile at 2’ position i.e. 2’-O-methyl or 2’- hydrogen protects the internucleotide bond from *in-line* cleavage.

The studies described above show that the full-length transcription products cannot be attained with 5’NH NTPs and the DNA-dependent RNA polymerases. There are several possible explanations for these results. The first explanation is that the 5’NH NTPs might not be substrates for the DNA-dependent RNA polymerases, and the second explanation is that the formed internucleotide phosphoramidate bond could be immediately cleaved due to the attack of the adjacent 2’-hydroxyl group, which causes the cleavage of internucleotide bond (Fig 2A) [9] or somehow combination of both. All that options may result in premature termination of transcription.

**Fig 3:**
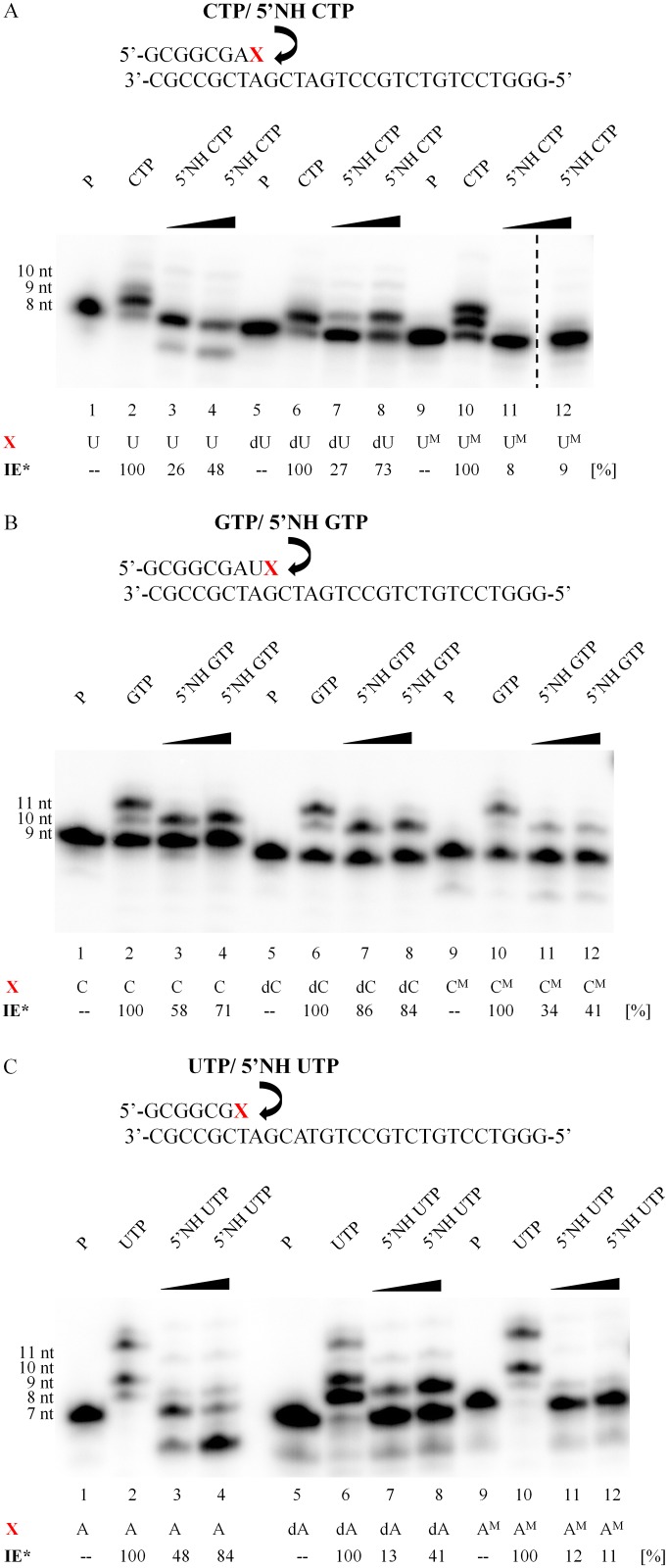
Primer-extension assay. Elongation complexes consisted of an unlabeled template DNA strand (bottom sequence) and a 5’-labeled RNA primer (top sequence). The X represents the last nucleotide in the RNA primer (the n-1 position), IE* represents the incorporation efficiency (normalized to a value of 100% for the natural rNTP reactions), and P represents non-elongated RNA primer. (A) The primer-extension assay conducted in the presence of the 5’NH CTP or natural CTP. (B) The primer-extension assay was conducted in the presence of 5’NH GTP or natural GTP. (C) The primer-extension assay was conducted in the presence of 5’NH UTP or natural UTP. The gels are a representative of three replicate experiments.

To determine the mechanism of the above phenomenon, a primer-extension assay originally developed by Pomerantz *et al*. was applied [26]. This approach consisted of a template DNA strand with an unpaired downstream region and RNA primers. The primers were designed in order to know in advance which ribonucleotide would be incorporated at the 3’-end of the transcript (called the “n” position). Furthermore, based on the mechanism of the phosphoramidate bond cleavage, the unmodified RNA primers as well as those containing 2’-deoxyribonucleotide and 2’-O-methyl ribonucleotide at the n-1 position (the 3’end of the primer) were used. In the primers with modified nucleotides, the 2’-hydroxyl group at the n-1 position of the oligonucleotide was modified to protect the internucleotide phosphoramidate bond between the n and n-1 nucleotides (formed during transcription reaction, Fig 2B). The extension reactions were performed in the standard transcription buffer, in the presence of only one type of ribonucleotide i.e. NTP or 5’NH NTP with the use of T7 RNA polymerase, which exhibited the highest ability to form intermediate products (Fig 1). The yields of the extension reactions for each set of primers were normalized to a value of 100% for the natural NTPs reactions (when more than one product was obtained, the transcription yield was the sum of all products). The analysis of Fig 3A–3C indicates that the presence of 2’-deoxyribonucleotide at the n-1 position increased the incorporation yield, whereas the presence of 2’-O-methyl ribonucleotides decreased its rate. Presumably, this unfavorable effect is due to steric hindrance caused by a methyl group, which is more bulky than the hydroxyl group. Additionally, the nature of the 5’NH NTPs influenced the merge efficiency and the highest rates of incorporation were obtained for 5’NH GTP. When cytidine, 2’-deoxycytidine or 2’-O-methyl cytidine were present at position n-1 of the primer, T7 RNA polymerase incorporated 5’NH GTP in 58, 86 and 34% yield, respectively (Fig 3B). The effectiveness of 5’NH CTP incorporation was 26, 27 and 8% when at position n-1 of the primer was uridine, thymidine and 2’-O-methyl uridine, respectively (Fig 3A). Using of 5’NH UTP results in 48, 13 and 12% incorporation yield when at the n-1 position of the primer was adenosine, 2’-deoxyadenosine and 2’-O-methyl adenosine, respectively (Fig 3C). The incorporation yield could be further improved by increasing the concentrations of the 5’NH NTPs. A similar set of incorporation experiments was also performed with 5’NH ATP and respective primers terminated at the 3’-end with guanosine, 2’-deoxyguanosine and 2’-O-methyl guanosine. Unfortunately, only cleavage products terminated with 2’,3’-cyclic phosphate could be observed (data not shown). This result suggests that 5’-N-triphosphates of 5’-amino-5’-deoxyadenosine, in contrast to 5’-N-triphosphates of 5’-amino-5’-deoxycytidine, uridine and guanosine, are not substrates for the T7 RNA polymerase.

Based on the incorporation results, we postulate that T7 RNA polymerase incorporates single 5’-N-triphosphates of 5’-amino-5’-deoxyguanosine, 5’-amino-5’-deoxycytidine, 5’-amino-5’-deoxyuridine but not 5’-N-triphosphate of 5’-amino-5’-deoxyadenosine into RNA strands. However, after incorporation, the internucleotide phosphoramidate bond is rapidly cleaved and the primer terminated with 2’,3’-cyclic phosphate and 5’-amino-5’-deoxyribonucleoside are released. Analysis of the gels shown in Fig 3A–3C indicates that when CTP, GTP or UTP were used in the extension reaction, the formation of oligonucleotides with multiple mergers of NTPs could be observed, especially in GTP (Fig 3B) and UTP (Fig 3C, line 2) pathways. Similar phenomenon was earlier reported by Kashkina *et al*. and could be caused by the incorporation of triphosphates of nucleosides into newly synthesized RNA strand based on non-canonical Watson-Crick base pairing [34] due to unbalanced substrate concentrations [35]. In the UTP reaction mixture the incorporation of four nucleotides was observed. The combination of misincorporation and misalignment due to looping out the DNA template could be potential explanation of this case [36]. What is more, it was previously shown that particularly G and U form thermodynamically stable mismatches [37, 38]. However, when 5’-N-triphosphates of 5’-amino-5’-deoxyribonucleosides were used in the extension reaction, only the incorporation of single nucleotide was observed (Fig 3A–3C, lanes 3, 4, 7, 8, 11 and 12). Moreover, in most cases, the major products of this reaction were oligonucleotides with faster gel mobility than the primers. It was shown that when unmodified RNA primer was used in the primer extension reaction, the products with fast gel mobility were likely oligonucleotides terminated with 2’,3’-cyclic phosphates, which were expected according to *in-line* cleavage of the internucleotide phosphoramidate bond. The primer extension reaction with various 5’-N-triphosphates of 5’-amino-5’-deoxyribonucleosides was performed several times and the results were reproducible; however, occasional differences were noted in the ratio of full-length product of transcription (primer with 5’NH-nucleotide) and 2’,3’-cyclic phosphate terminated primer were observed. The most stable primer extension reaction product was 3’-terminated oligonucleotide with 5’NH-guanosine; however, the 2’,3’-cyclic phosphate terminated primer could also be observed. Treatment of the reaction mixture with 5% acetic acid resulted in the cleavage of primer-5’NH-gaunosine oligonucleotide (S4 Fig) [9]. When the reaction mixture contained the 5’-N-triphosphates of 5’-amino-5’-deoxycytidine or 5’-amino-5’-deoxyuridine, the major reaction products were almost exclusively 2’,3’-cyclic phosphate-terminated primers. The introduction of 2’-deoxyribonucleotides or 2’-O-methyl ribonucleotides into the n-1 position of the RNA primer prevents the transcription products containing at 3’-side 5’NH-nucleotide from *in-line* cleavage. In consequence, the transcription products were stable and the cleavage of internucleotide phosphoramidate bond was not observed.

In conclusion, it was demonstrated that the application of 5’-N-triphosphates of 5’-amino-5’-deoxyribonucleosides dramatically lowered the rates of transcription. Taking these properties into account, it could be postulated that 5’NH NTPs can be used as potential transcription inhibitors. Our findings expand the knowledge on the suitability of the 5’-N-triphosphates of 5’-amino-5’-deoxyribonucleosides and the exact mechanism of transcriptional inhibition. Additionally, the presented studies provide basic information on the process of in line cleavage of internucleotide phosphoramidate bond in RNA oligonucleotides. The implementation of the above experiments are new and original developments in the area of 5’NH NTP use in biological research.

## Supporting Information

S1 Fig HPLC chromatograms. (A) HPLC chromatogram of 5’NH ATP. (B) HPLC chromatogram of 5’NH CTP. (C) HPLC chromatogram of 5’NH GTP. (D) HPLC chromatogram of 5’NH UTP. Chromatography was performed on a 1260 Infinity LC System (Agilent Technologies). Separation was achieved with a X Terra^®^ Prep RP18 column, 7.8 × 150 mm, having a particle size of 7 μm (Waters). The column was kept at 25°C during analysis, and injection volume was 50 μl. Mobile phase A was 100 mM triethylammonium bicarbonate in Milli-q water and mobile phase B was 100 mM triethylammonium bicarbonate in 40% acetonitrile (ACN). The gradient elution was performed as follows: 0 min– 100% A, 0% B, flow 2 mL/min; 30 min– 50% A, 50% B, flow 2 mL/min; 32 min– 100% A, 0% B, flow 2 mL/min; 38 min– 100% A, 0% B, flow 2 mL/min.
(TIF)

S2 Fig T4 PNK phosphorylation reaction. Lane 1 –non-phosphorylated RNA primer (P). Lane 2 –T4 PNK phosphorylation carried out with ATP. Lane 3 –T4 PNK phosphorylation carried out with GTP. Lane 4 –T4 PNK phosphorylation carried out with 5’NH ATP. Lane 5 –T4 PNK phosphorylation carried out with 5’NH CTP. Lane 6 –T4 PNK phosphorylation carried out with 5’NH GTP. Lane 7 –T4 PNK phosphorylation carried out with 5’NH UTP.
(TIF)

S3 Fig Transcription reaction. (A) Transcription with 5S rRNA DNA templates using RNAP T3 and standard transcription buffer (lanes 1–7), BT1 buffer (lanes 8–11), BT2 buffer (lanes 12–15), and BT3 buffer (lanes 16–19). Lanes 1, 8, 12, 16 –control reactions. Lanes 5, 9, 13, 17 –reactions with 5’NH CTP. Lanes 6, 10, 14, 18 –reactions with 5’NH GTP. Lanes 7, 11, 15, 19 –reactions with 5’NH UTP. Lanes 2, 3, 4 –reactions without CTP, GTP, or UTP, respectively. (B) Transcription with 5S rRNA DNA templates using natural RNAP Sp6 standard transcription buffer (lanes 1–4), BT1 buffer (lanes 5–8), BT2 buffer (lanes 9–12), and BT3 buffer (lanes 13–16). Lanes 1, 5, 9, 13 –control reactions. Lanes 2, 6, 10, 14 –reactions with 5’NH CTP. Lanes 3, 7, 11, 15 –reactions with 5’NH GTP. Lanes 4, 8, 12, 16 –reactions with 5’NH UTP. (C) Transcription with 5S rRNA DNA templates using natural RNAP T7 standard transcription buffer (lanes 1–4), BT1 buffer (lanes 5–8), BT2 buffer (lanes 9–12), BT3 buffer (lanes 13–16). Lanes 1, 5, 9, 13 –control reactions. Lanes 2, 6, 10, 14 –reactions with 5’NH CTP. Lanes 3, 7, 11, 15 –reactions with 5’NH GTP. Lanes 4, 8, 12, 16 –reactions with 5’NH UTP. (D) Transcription with 5S rRNA DNA templates using natural RNAP T7 Y639F standard transcription buffer (lanes 1–4), BT1 buffer (lanes 5–8), BT2 buffer (lanes 9–12). Lanes 1, 5, 9 –control reactions. Lanes 2, 6, 10 –reactions with 5’NH CTP. Lanes 3, 7, 11 –reactions with 5’NH GTP. Lanes 4, 8, 12 –reactions with 5’NH UTP. (E) Transcription with 5S rRNA DNA templates using natural RNAP T7 (lanes 1–4) or RNAP T7 Y639F (lanes 5–8) and standard BT4. Lanes 1, 4 –control reactions. Lanes 2, 6 –reactions with 5’NH CTP. Lanes 3, 7 –reactions with 5’NH GTP. Lanes 4, 8 –reactions with 5’NH UTP. The gels are a representative of three replicate experiments.
(TIF)

S4 Fig Chemical cleavage of primer-extension reaction product. Lane 1 –non-elongated RNA primer. Lane 2 –primer extension reaction carried out with GTP. Lane 3 –primer extension reaction carried out with GTP, followed by treatment with 5% acetic acid at 37°C for 20 min. Lane 4 –primer extension reaction carried out with GTP, followed by treatment with 0.1 N hydrochloric acid at 30°C for 20 min. Lane 5 –primer extension reaction carried out with 5’NH GTP. Lane 6 –primer extension reaction carried out with 5’NH GTP, followed by treatment with 5% acetic acid at 37°C for 20 min. Lane 7 –primer extension reaction carried out with 5’NH GTP, followed by treatment with 0.1 N hydrochloric acid at 30°C for 20 min. The gels are a representative of three replicate experiments.
(TIF)

S1 Table Oligonucleotide sequences employed in transcription reaction (coding 5S rRNA). (DOCX)

## Abbreviations

5’NH NTPs: 5’-N-triphosphates of 5’-amino-5’-deoxyribonucleosides
RNAP T7: T7 RNA polymerase
RNAP T3: T3 RNA polymerase
RNAP Sp6: Sp6 RNA polymerase
TLC: thin-layer chromatography
HPLC: high-performance liquid chromatography
