# Supplementary figures and images for: Studies on Transcriptional Incorporation of 5’-N-Triphosphates of 5’-Amino-5’-Deoxyribonucleosides
Source: PLoS One. 2016 Feb 1;11(2):e0148282. doi: 10.1371/journal.pone.0148282 (PMC4735469; doi:10.1371/journal.pone.0148282)

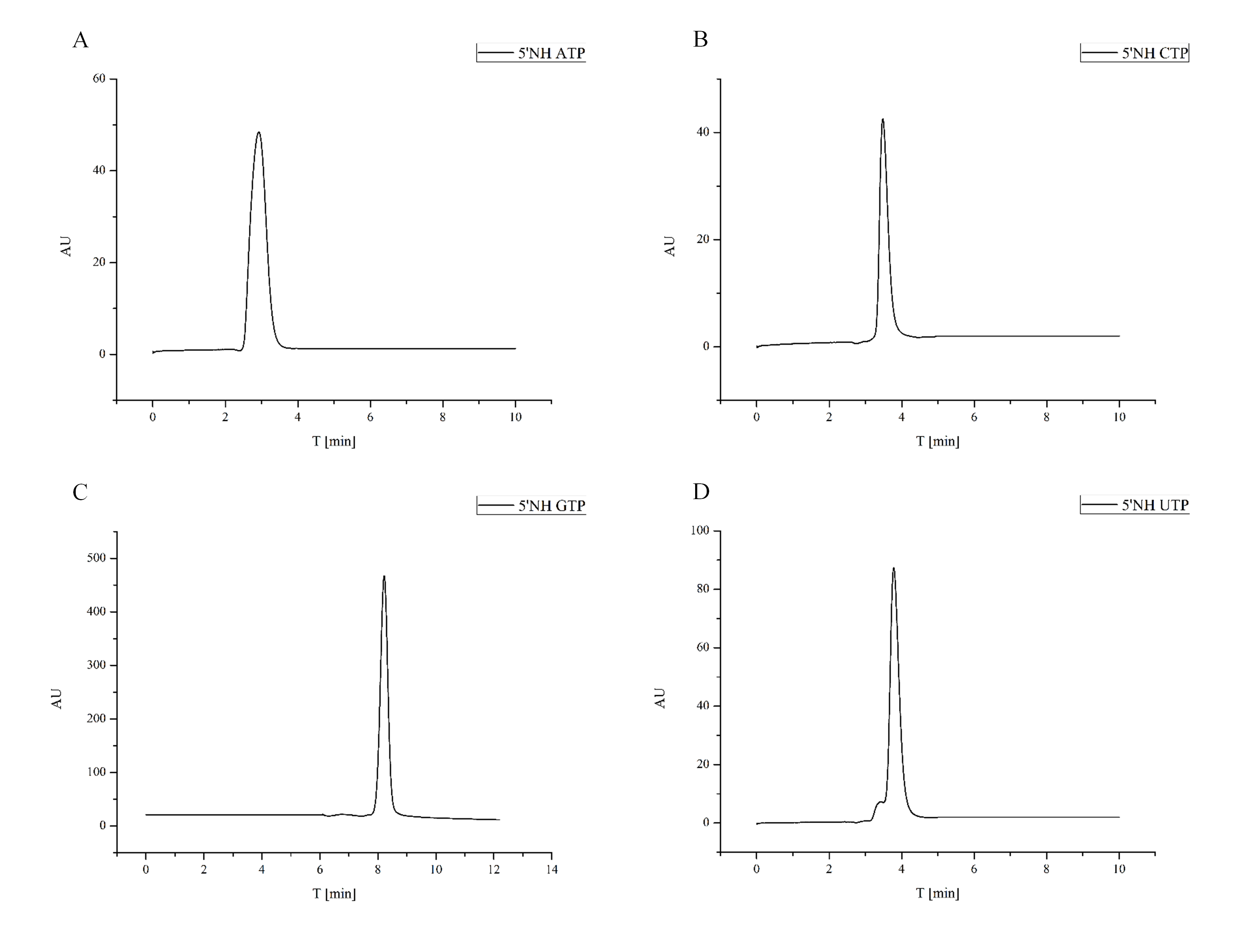

Supplement: S1 Fig — (A) HPLC chromatogram of 5’NH ATP. (B) HPLC chromatogram of 5’NH CTP. (C) HPLC chromatogram of 5’NH GTP. (D) HPLC chromatogram of 5’NH UTP. Chromatography was performed on a 1260 Infinity LC System (Agilent Technologies). Separation was achieved with a X Terra® Prep RP18 column, 7.8 × 150 mm, having a particle size of 7 μm (Waters). The column was kept at 25°C during analysis, and injection volume was 50 μl. Mobile phase A was 100 mM triethylammonium bicarbonate in Milli-q water and mobile phase B was 100 mM triethylammonium bicarbonate in 40% acetonitrile (ACN). The gradient elution was performed as follows: 0 min– 100% A, 0% B, flow 2 mL/min; 30 min– 50% A, 50% B, flow 2 mL/min; 32 min– 100% A, 0% B, flow 2 mL/min; 38 min– 100% A, 0% B, flow 2 mL/min. (TIF) [file pone.0148282.s001.tif]

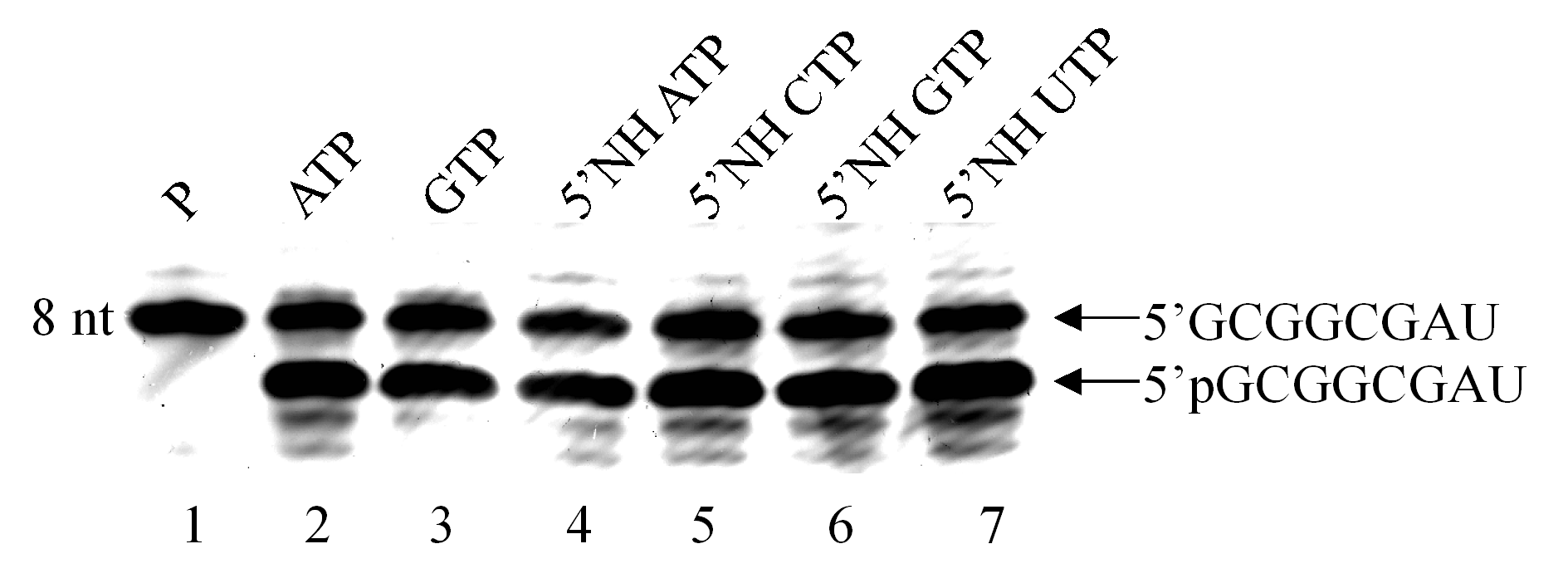

Supplement: S2 Fig — Lane 1 –non-phosphorylated RNA primer (P). Lane 2 –T4 PNK phosphorylation carried out with ATP. Lane 3 –T4 PNK phosphorylation carried out with GTP. Lane 4 –T4 PNK phosphorylation carried out with 5’NH ATP. Lane 5 –T4 PNK phosphorylation carried out with 5’NH CTP. Lane 6 –T4 PNK phosphorylation carried out with 5’NH GTP. Lane 7 –T4 PNK phosphorylation carried out with 5’NH UTP. (TIF) [file pone.0148282.s002.tif]

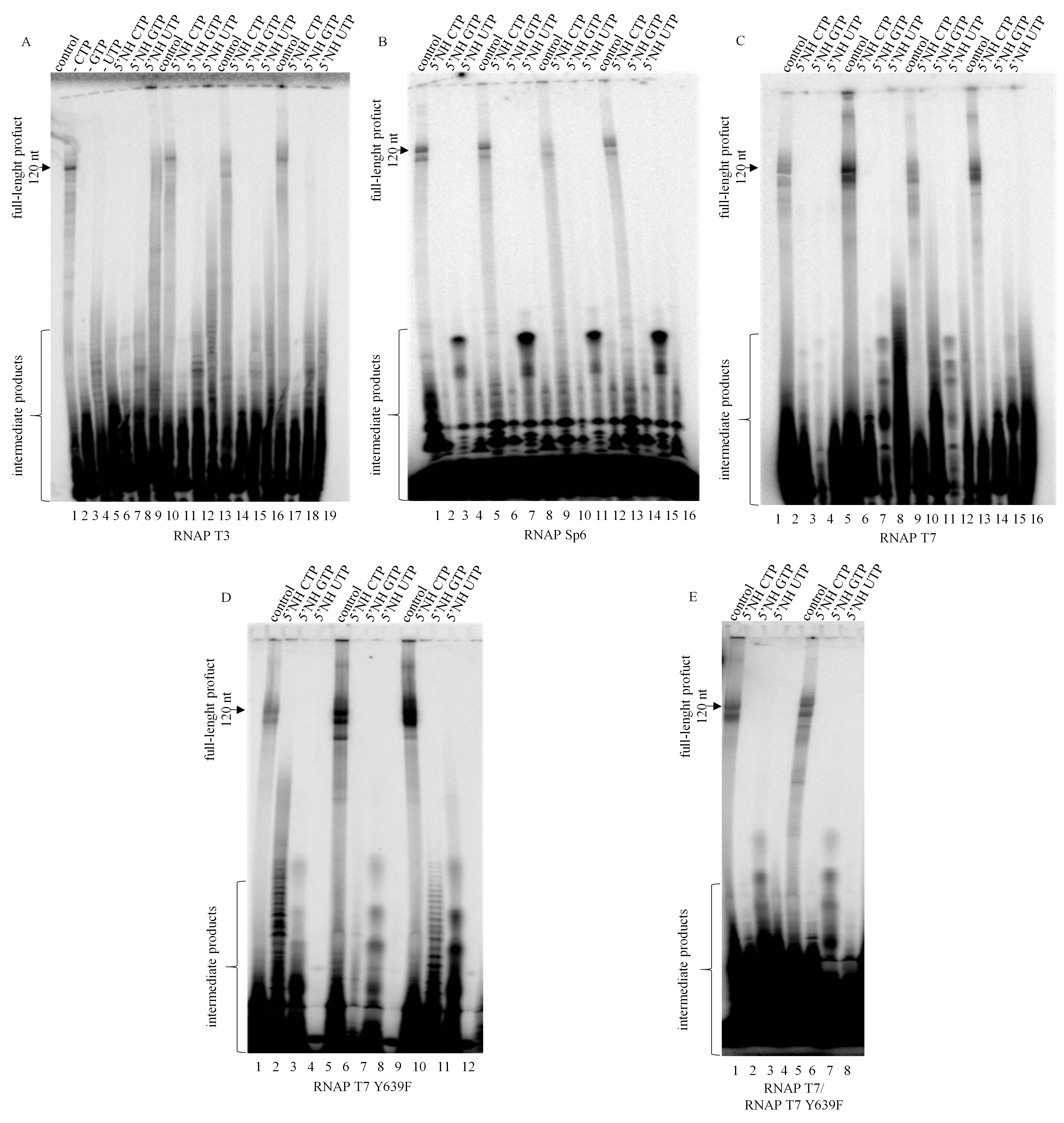

Supplement: S3 Fig — (A) Transcription with 5S rRNA DNA templates using RNAP T3 and standard transcription buffer (lanes 1–7), BT1 buffer (lanes 8–11), BT2 buffer (lanes 12–15), and BT3 buffer (lanes 16–19). Lanes 1, 8, 12, 16 –control reactions. Lanes 5, 9, 13, 17 –reactions with 5’NH CTP. Lanes 6, 10, 14, 18 –reactions with 5’NH GTP. Lanes 7, 11, 15, 19 –reactions with 5’NH UTP. Lanes 2, 3, 4 –reactions without CTP, GTP, or UTP, respectively. (B) Transcription with 5S rRNA DNA templates using natural RNAP Sp6 standard transcription buffer (lanes 1–4), BT1 buffer (lanes 5–8), BT2 buffer (lanes 9–12), and BT3 buffer (lanes 13–16). Lanes 1, 5, 9, 13 –control reactions. Lanes 2, 6, 10, 14 –reactions with 5’NH CTP. Lanes 3, 7, 11, 15 –reactions with 5’NH GTP. Lanes 4, 8, 12, 16 –reactions with 5’NH UTP. (C) Transcription with 5S rRNA DNA templates using natural RNAP T7 standard transcription buffer (lanes 1–4), BT1 buffer (lanes 5–8), BT2 buffer (lanes 9–12), BT3 buffer (lanes 13–16). Lanes 1, 5, 9, 13 –control reactions. Lanes 2, 6, 10, 14 –reactions with 5’NH CTP. Lanes 3, 7, 11, 15 –reactions with 5’NH GTP. Lanes 4, 8, 12, 16 –reactions with 5’NH UTP. (D) Transcription with 5S rRNA DNA templates using natural RNAP T7 Y639F standard transcription buffer (lanes 1–4), BT1 buffer (lanes 5–8), BT2 buffer (lanes 9–12). Lanes 1, 5, 9 –control reactions. Lanes 2, 6, 10 –reactions with 5’NH CTP. Lanes 3, 7, 11 –reactions with 5’NH GTP. Lanes 4, 8, 12 –reactions with 5’NH UTP. (E) Transcription with 5S rRNA DNA templates using natural RNAP T7 (lanes 1–4) or RNAP T7 Y639F (lanes 5–8) and standard BT4. Lanes 1, 4 –control reactions. Lanes 2, 6 –reactions with 5’NH CTP. Lanes 3, 7 –reactions with 5’NH GTP. Lanes 4, 8 –reactions with 5’NH UTP. The gels are a representative of three replicate experiments. (TIF) [file pone.0148282.s003.tif]
