# Supplementary material for: Studies on Transcriptional Incorporation of 5’-N-Triphosphates of 5’-Amino-5’-Deoxyribonucleosides
Source: PLoS One. 2016 Feb 1;11(2):e0148282. doi: 10.1371/journal.pone.0148282 (PMC4735469; doi:10.1371/journal.pone.0148282)
Supplement: S1 Table — (DOCX) [file pone.0148282.s005.docx]

| **Name** | **Sequence** |
| --- | --- |
| **O1** | GCTAGAATTCTAATACGACTCACTATAGGGTGCCTGGCGGCAGTAGCGCGGTGGTCCCACCTGACCCCATGCCGAACTCAGAAGTGAAACGCCGTAGCGCCGATGGTAGTGTGGGGTCTCCCCATGCGAGAGTAGGGAACTGCCAGGCATCTGCAGTACG |
| **O2** | CGTACTGCAGATGCCTGGCAGTTCCCTACTCTCGCATGGGGAGACCCCACACTACCATCGGCGCTACGGCGTTTCACTTCTGAGTTCGGCATGGGGTCAGGTGGGACCACCGCGCTACTGCCGCCAGGCACCCTATAGTGAGTCGTATTAGAATTCTAGC |
